# A case report of endocarditis and spondylitis caused by *Brucella melitensis* biovar 3

**DOI:** 10.1186/s12879-021-06142-3

**Published:** 2021-05-20

**Authors:** Huan Zhang, Songsong Xie, Yueli Wang, Xiaoli Zhao, Jihai Yi, Zhen Wang, Qi Liu, Xiaoyu Deng, Bingjie Li, Buyun Cui, Yuanzhi Wang, Chuangfu Chen

**Affiliations:** 1 School of Animal Science and Technology, Shihezi University, Shihezi City, 832000 Xinjiang China; 2 NHC Key Laboratory of Prevention and Treatment of Central Asia High Incidence Diseases, Shihezi City, 832000 Xinjiang China; 3 The First Affiliated Hospital of Shihezi University, Shihezi City, 832000 Xinjiang China; 4 State Key Laboratory for Infectious Disease Prevention and Control, Collaborative Innovation Center for Diagnosis and Treatment of Infectious Diseases, National Institute for Communicable Disease Control and Prevention, Chinese Center for Disease Prevention and Control, Beijing, 100050 China; 5 School of Medicine, Shihezi University, Shihezi City, 832000 Xinjiang China

**Keywords:** *Brucella melitensis biovar* 3, Endocarditis, Spondylitis, Shepherd

## Abstract

**Background:**

This case report describes the clinical process of a shepherd who suffered brucellosis-related endocarditis (BE) and spondylitis (BS) and was infected with *Brucella melitensis biovar* 3 (*B. melitensis biovar* 3).

**Case presentation:**

A 55-year-old male patient was admitted to The First Affiliated Hospital of Shihezi University on October 11, 2018, due to over 3 months of intermittent fever, back pain, and heart trouble. The Rose Bengal Plate test was positive, the standard agglutination test titer for brucellosis was 1/800, and the blood culture was positive for *B*. *melitensis biovar* 3. Three instances of transthoracic echocardiography examination at days 1, 25, and 376 after admission to the hospital and magnetic resonance imaging (MRI) and computed tomography (CT) checks at days 5 and 38 revealed that the size of the vegetation on the posterior leaflet of the mitral valve increased from 0.71.4cm to 1.21.5cm and that the left atrium and ventricle were enlarged. The MRI and CT results showed hyperplasia of the second and third vertebra, a cold abscess formed on both sides of the psoas major muscles, and the vertebra hyperplasia became aggravated at a later time point. The patients situation deteriorated, and heart failure was discovered on October 22, 2019. At the moment of submission of this manuscript, the patient remains in bed at home because of severe debility caused by brucellosis.

**Conclusions:**

This is the first reported case of endocarditis combined with spondylitis caused by *B*. *melitensis biovar* 3 in a shepherd. Brucellosis infection can cause work-power losses because of misdiagnosis or a lack of proper treatment. Early diagnosis and treatment are essential for a successful outcome.

**Supplementary Information:**

The online version contains supplementary material available at 10.1186/s12879-021-06142-3.

## Background

Brucellosis is a worldwide zoonotic disease caused by *Brucella* spp. Humans can be infected by *Brucella* mainly through the consumption of unpasteurized dairy products, inhalation of infected aerosolized particles, and close contact with infected animals [1]. *Brucella* infection causes lesions in multiple organs in the human body. The spleen, liver, testis, bone marrow, and reticuloendothelial cells are the most affected, while cardiovascular and osteoarticular involvements, such as endocarditis, myocarditis, and spondylitis, are rare [2]. A total of 44,036 brucellosis cases occurred in China in 2019 according to the latest data released by the China Centers for Disease Control and Prevention (http://www.chinacdc.cn). The Xinjiang Uygur Autonomous Region (XUAR) is one of the leading pastoral areas in China, which is also an epidemic area with high morbidity due to brucellosis [3]. However, only a few studies regarding endocarditis and spondylitis attributable to *Brucella* have been reported in China, especially in remote regions. This study focuses on a patient living in the XUAR who suffered from brucellosis-related endocarditis (BE) and spondylitis (BS). The manifestations in this patient are described, and the genetic characteristics of the pathogenic agent are analyzed.

## Case presentation

**Fig. 1:**
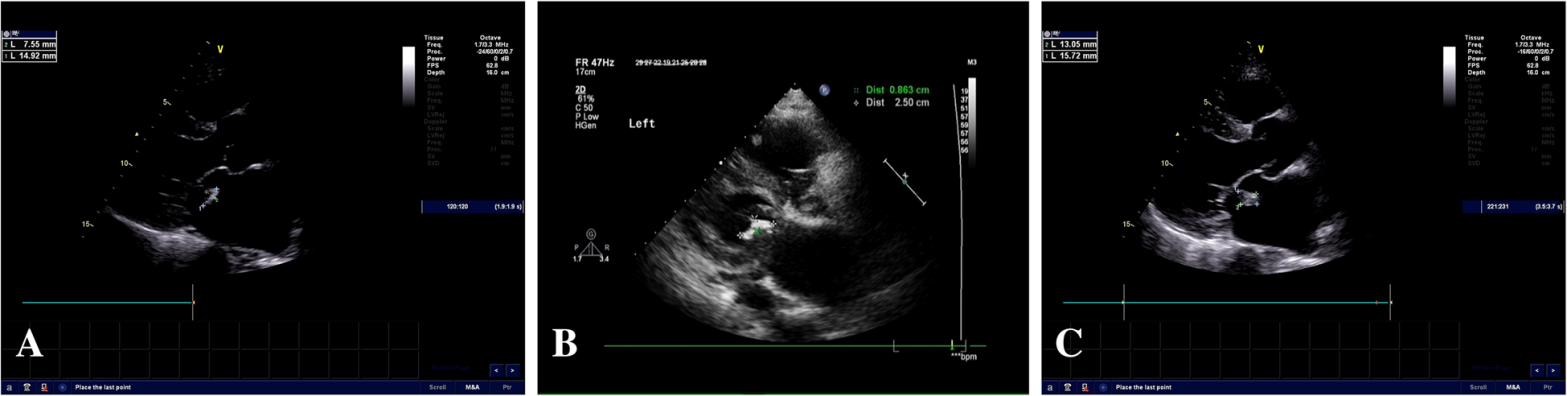
Transesophageal echocardiography showing the vegetation (red arrow) on the posterior leaflet of the mitral valve at different times. **a** October 11, 2018; **b** November 6, 2018; **c** October 22, 2019

**Fig. 2:**
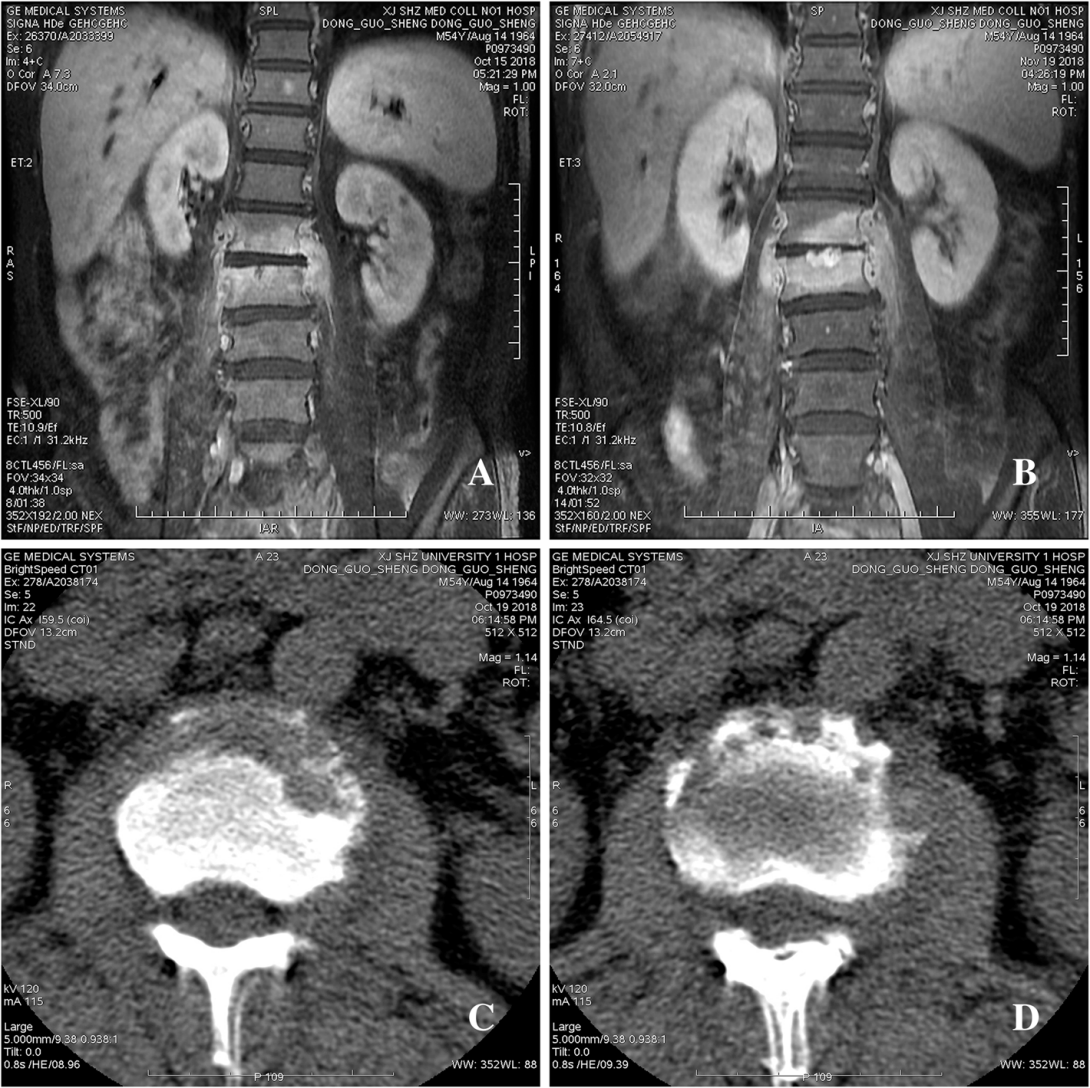
The patient with *Brucella* spondylitis at L23. **ad** The MRI and CT examinations showed the destruction of the vertebral bodies of L23 at different times. **a**, **b** The MRI examination on October 15 and November 19, 2018. **c**, **d** The CT examination on October 15 and November 19, 2018. The red arrows indicate abscess-formed area, and the green arrows indicate the hyperplasia-formed area

A 55-year-old male shepherd without preexisting diseases began to experience an intermittent fever up to 3839C and back pain on July 1, 2018. The patient was started on oral cephalosporin for 3 months (self-administered), but his symptoms persisted. The patient was sent to the First Affiliated Hospital of Shihezi University with a normal body temperature at 36.6C, where he underwent transthoracic echocardiography (TTE) on October 11, 2018. Vegetation (0.71.5cm) was discovered on the posterior leaflet of the mitral valve by a TTE examination (Fig.1a), which caused a mild regurgitation. The serum tube agglutination test (SAT) for *Brucella* was positive with a titer of 1:800, but the culture result was negative. The blood test results showed that the hemoglobin levels were reduced to 120g/L (see Additionalfile1). The levels of platelets, RBC, and albumin were lower than the normal range. However, the levels of globulin and erythrocyte sedimentation rate (ESR) were higher than the normal range (see Additionalfile 1). A combination of treatment with doxycycline (200mg/day) and rifampicin (900mg/day) was administered to the patient. However, the clinical symptoms were not relieved, and the patient was experiencing a serious backache. The MRI examination revealed that a cold abscess had formed on both sides of the psoas major muscles and the left side of the erector spinae muscle (Fig.2a), and the CT results indicated hyperplasia of the second and third vertebra (Fig. 2c) on October 15, 2018. The MRI showed a low signal intensity on the T1-weighted images and a high signal intensity on the T2-weighted images of the second and third vertebra. Because of financial constraints, the patient refused to undergo surgical treatment. Hence, he was prescribed oral doxycycline (100mg/dose, twice a day) and rifampicin (600mg/dose, once daily) as recommended by the World Health Organization.

Because of a lack of timely medical follow-up, on November 6, 2018, the patients condition deteriorated, the size of the vegetation increased to 0.82.5cm (Fig. 1b) along with a hemorrhage that occurred on the gingiva, and a hemorrhagic spot formed on both lower limbs. An echocardiogram confirmed the presence of severe regurgitation. Additionally, the left atrium and ventricle were enlarged, and the left ventricular ejection fraction was 64%. The blood test results showed that the platelet level dropped rapidly over time, reaching its lowest level at 2910^9^/L on November 6, 2018 (see Additionalfile 1). The MRI examination showed that both the second and third vertebra hyperplasia and abscess in the muscle increased significantly on November 19, 2018 (Fig. 2b, d). The antibiotics for brucellosis were changed to levofloxacin (400mg/dose, once daily), doxycycline (100mg/dose, twice a day), and sulfamethoxazole tablets (600mg/dose, once daily), and the patient was treated with medicines to enhance the platelet levels at the same time.

**Fig. 3:**
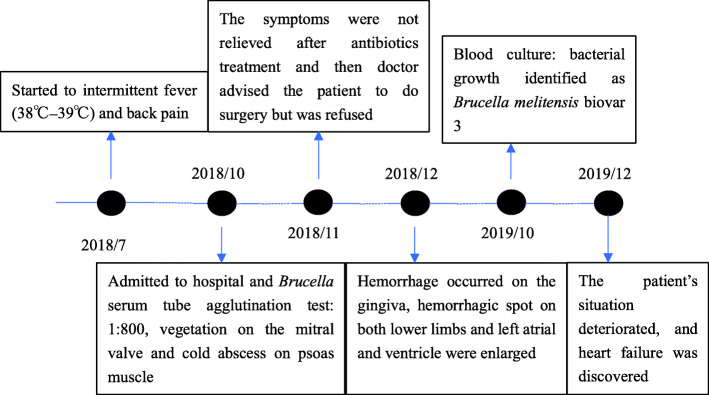
The onset and outcome of the disease, diagnosis, and treatment

However, the patients situation deteriorated again, and heart failure was discovered on October 22, 2019. The size of the vegetation was 1.31.5cm (Fig. 1c). The left atrium and ventricle were further enlarged, and the left ventricular ejection fraction was 58%. The end-diastolic volume (EDV) and end-systolic volume (ESV) of the left ventricular were 304ml and 105ml, respectively. A strong regurgitation signal presented on the mitral valve, with a pulse rate of 67 beats/min. The fraction shortening (FS) value was 36%. Although the platelet level had recovered to 5210^9^/L, the hemoglobin level sharply decreased to 85g/L (see Additionalfile 1). The NT-proBNP levels reached 4152.0pg/mL, 33 times higher than the normal level. It is worth noting that the level of creatinine was normal, except on October 22, 2019, when it reached its peak level at 252.6 umol/L (see Additionalfile 1). Additionally, the bone marrow aspiration result showed that the proliferation of bone marrow was considerably reduced, the granulocyte and erythrocytes were multiplicative, and the proportion of neutrophilic segmented granulocytes increased with the development of granulocyte lineage hyperplasia in the bone marrow (data not shown). At the moment of submission of this manuscript, the patient remains in bed at home because of severe debility caused by brucellosis. Now, the patient must undergo regular renal dialysis as a follow-up, and on June 26, 2020, the patient had renal dialysis. The clinical symptoms and diagnostic results at different times are listed in Additionalfile 1. The onset, diagnosis, and treatment of the disease in this patient are shown in Fig.3.

### Serological tests

The diagnosis of brucellosis was based on the rose bengal plate test (RBPT) and the SAT. The RBPT and SAT *Brucella* antigen were purchased from the Institute of Infectious Disease of the China Centers for Disease Control and Prevention. The SAT result was 1:800.

### Pathogen isolation and identification

Five milliliters of venous blood was collected from the patient on October 11, 2018 and October 22, 2019, and the blood samples were injected into a biphase blood culture and incubated at 37C for 5 days. Conventional biological methods were used for the isolation and identification of the bacteria [4]. The minor phenotypic differences were used to distinguish the biovars of *Brucella*, including serotyping, phage typing, fuchsin and thionin dye sensitivity, CO_2_ requirement, H_2_S production, and metabolic properties, and *Brucella melitensis* 16M was used as the reference strain. This process was completed at the Brucellosis Laboratory, the National Institute for Communicable Disease Control, and the Center for Disease Prevention and Control (CDC) of China in Beijing.

The specific sequences of the IS*711* primers have been described in previous work [5]. The reaction system for the gene sequencing included 13L ddH_2_O, 15L master mix, 0.5L of each primer, and 1.5L of the DNA template. The amplification conditions were as follows: 95C for 5min, 30cycles at 95C for 2min, 55C for 2min, 72C for 2min, and a final incubation at 72C for 4min. The positive PCR products were purified using the TIAN-gel Mini Purification Kit (TIANGEN, Beijing, China) and sequenced by Sangon Biotech Co., Ltd. (Shanghai, China). R version 3.6.1 was used to construct the tree according to the packages ggplot2, ggtree [6], and colorspace. The DNA extractions were performed using a whole bacterial genome nucleic acid extraction kit [Tiangen Biotech (Beijing) Co., Ltd., Beijing, China]. The IS*711* primers were synthesized by the Sangon Biotech Co., Ltd. (Beijing, China).

## Results

### Bacterial isolation and identification

The colonies of bacteria isolated from the patients blood were semitransparent and round in shape, with smooth surfaces. Conventional biological identification showed the colonies to be short Gram-negative bacilli that did not produce H_2_S and CO_2_. The urea, basic fuchsin, and thionin tests were positive. The A and M monospecific antisera agglutination and the bacteriophage BK_2_ test were positive. The Tb, Wb, and R/C phage typing tests were negative, suggesting that the colonies corresponded to the *B. melitensis* biovar 3, an endemic strain in the XUAR [5, 7].

### Phylogenetic analysis

**Fig. 4:**
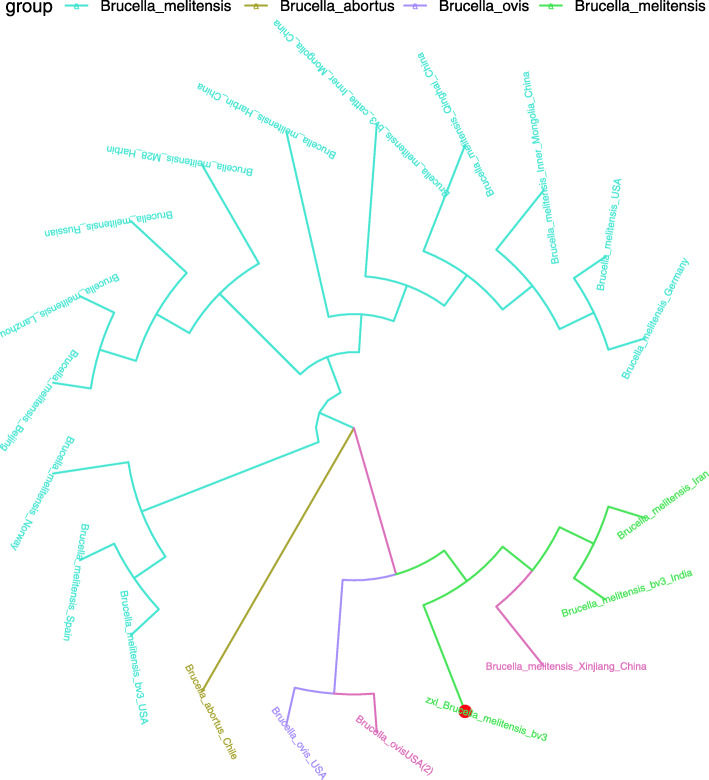
The phylogenetic tree of the IS*711* concatenated sequence of *Brucella melitensis* (red circle) isolated from human blood in this study and the reference sequences from *Brucella melitensis* retrieved from the GenBank database

A phylogenetic tree was constructed based on the 731bp sequence of the IS*711* repetitive element for the isolate. The nucleotide sequence from this study was deposited in the GeneBank database (IS*711*: MT846927). The phylogenetic analysis showed that the *Brucella* isolates in this study closely matched those of *B. melitensis* biovar 3 isolated from the Asia badger in Xinjiang, China (Fig.4) [8].

## Discussion and conclusions

Brucellosis is a worldwide zoonosis and systemic disease. The transmission to humans occurs primarily through contact with infected animals or the consumption of contaminated food such as raw milk and its byproduct [9]. *Brucella* infection cause lesions in any organ and system in the human body. The clinical symptoms of brucellosis include intermittent fever, night sweats, and joint and muscle pain [10]. The complications caused by brucellosis include osteoarticular, epididymal orchitis, nervous disorders, and liver involvement [11]. However, endocarditis combined spondylitis caused by *Brucella* is rarely reported. The incidence rate of endocarditis accounts for approximately only 2% of all brucellosis cases, but it is responsible for 80% of brucellosis-related mortality [12]. Jia et al. [13] studied 10 BE cases and discovered a high death rate without the replacement of valves. In contrast, all of those who accepted prosthetic valve replacement surgery combined with at least 6 weeks of drug treatment survived and showed no relapse. Dourakis et al. [14] found that the implantation of the defibrillator itself could induce a case of BE. The patient recovered after the defibrillator was removed, and the pacemaker was explanted. In addition, the large vegetation was removed through surgery. Mahjoub et al. [15] studied infective endocarditis caused by *Brucella*. The patient showed a good prognosis after receiving a high dose of antibiotics and underwent surgery with mitral valve replacement. It has been reported that the mortality rate of BE treated with a combination of antibiotics and surgery was 6.7%, whereas that with antibiotic treatment only was 32.7% [16]. In our case report, the patient suffered brucellosis-induced endocarditis and spondylitis. However, because of financial constraints, the patient refused to undergo surgical treatment that might have improved his condition. Intriguingly, vegetation has been found in most BE cases. It has been reported that *Brucella* can often cause valvular lesions and cardiac insufficiency [17]. The reason why *Brucella* can cause vegetation on the valve is unknown.

Brucellosis can affect the entire vertebral column, and the lumbar spine is the most frequently involved vertebral region, followed by the thoracic and cervical segments [18, 19]. It is well known that the multiple and contagious involvement of vertebral bodies occurs in tuberculous spondylodiscitis [20]. However, *Brucellas* involvement in numerous vertebral bodies has been described in only 920% cases [21]. The infection not only affects the vertebrae but also causes damage to neighboring tissues, including epidural and para-prevertebral tissues, psoas muscles, and radicles. Ulu-Kilic and colleagues [18] studied 293 patients with spinal brucellosis in Turkey, and psoas abscess only accounted for 3.4% (10) patients. In this study, hyperplasia occurred on the patients second and third centrum, and a cold abscess formed on both sides of the psoas major muscles and the left side of erector spinae muscle. The thick abscess that formed in the psoas and spinae muscle suggested complicated BS according to the definitions [18].

The most common manifestations among BE or patients with BS are fever, sweating, backache, debility, spleen, and liver enlargement [17, 18]. In this study, in addition to the symptoms mentioned previously, the patient suffered a hemorrhage on the gingiva, a hemorrhagic spot formed on the lower limbs, and even edema appeared when heart failure was discovered on October 22, 2019. Very few studies have reported such clinical symptoms before. Hence, it could be an atypical manifestation for the patient who suffered BE combined with BS.

*B. abortus* and *B. melitensis* are the most frequently isolated species from BE patients, but *Brucella suis* is rarely found (5% of cases) [15, 17, 22, 23]. *B. melitensis* causes more severe diseases associated with disabling complications [24]. Of note, *B. melitensis* is also the most predominant pathogen isolated from BS cases, with 61% biovar 3 isolates and 39% biovar 1 isolate [18, 25,27]. In this study, the organism was also identified as *B. melitensis* biovar 3, and this was consistent with previous studies where human and livestock brucellosis were primarily caused by *B. melitensis* biovar 3 in XUAR [5, 28]. Interestingly, the *Brucella* isolates in this study closely matched the *B. melitensis* biovar 3 isolated from the Asia badger, which indicates that the Asia badger is a *Brucella* spillover host that infects sheep or cattle and then acts as a reservoir host, and this poses a massive threat to livestock and humans [8].

For those with brucellosis endocarditis and spondylitis, drug treatment combined with surgery is the best approach, especially for patients with severe endocarditis and spondylitis. However, surgery is the last option for treating BS [29]. Maryam [16] reported that the mortality rate of BE with drugs combined with surgical treatment was 6.7%, but the drug treatment solely was 32.7%. There is no consensus for treating BE and BS. Patients are typically given doxycycline and rifampicin with or without aminoglycoside [30, 31].

Alternatively, it has been reported that streptomycin combined with doxycycline has superior efficacy and lower relapse rates than other treatments [27, 32]. The duration of antibiotic therapy in patients is adjusted according to the clinical manifestation. In addition, financial constraints, health care system-related differences, and follow-up are important factors that affect a patients treatment. In this study, the patient was not a local resident and his medical insurance did not cover the Shihezi health care system. Hence, the local government could only provide a partial subsidy for the patients medical costs according to health care system regulations. However, the patient still refused to undergo surgery due to financial constraints. In addition, timely follow-up was not able to be obtained, which might be another reason for the deterioration of the patients condition. These situations may be present for some patients; hence, they cannot be ignored in the treatment.

In summary, this is the first reported case of endocarditis combined with spondylitis caused by *B. melitensis* biovar 3 isolated from a shepherd in China. Brucellosis infection can cause work-power losses because of misdiagnosis or lack of proper treatment. Although there is no standard therapy protocol for treating BE and BS, early diagnosis and treatment are essential for a successful outcome.

## Supplementary Information

**Additional file 1: Table S1**. Clinical and laboratory data of the patient with *Brucella* endocarditis and spondylitis.

## Data Availability

All of the data generated or analyzed in this study are included in this published article.
