# Supplementary material for: A case report of endocarditis and spondylitis caused by Brucella melitensis biovar 3
Source: BMC Infect Dis. 2021 May 20;21:460. doi: 10.1186/s12879-021-06142-3 (PMC8139066; doi:10.1186/s12879-021-06142-3)
Supplement: Supplementary file 1 — Additional file 1: Table S1. Clinical and laboratory data of the patient with Brucella endocarditis and spondylitis. [file 12879_2021_6142_MOESM1_ESM.pdf]

Table S1. Clinical and laboratory data of the patient with *Brucella* endocarditis and spondylitis.

Characteristics	Date				
	2018/7/1	2018/10/11	2018/10/27	2018/11/6	2019/10/22
**Symptoms and signs**					
Fever	+	+	+	+	+
Debility	+	+	+	+	+
Sweating	+	+	+	+	+
Arthralgia	+	+	+	+	+
Backache	+	+	+	+	+
Gingiva hemorrhage				+	+
Hemorrhagic spot on lower limbs				+	+
Hepatomegaly					+
Splenomegaly					+
Edema					+
**Diagnostic results**					
SAT (titer)	NA	1/800	NA	NA	1/800
Cold abscess formed in psoas/spinae muscle	NA	+	NA	++	-
Vegetations (size)	NA	0.7*1.5cm	NA	0.8*2.5cm	1.3*1.5cm
Enlarged left atrial/ventricle	NA	-	NA	+	++
Heart failure	NA	-	NA	-	+
**Laboratory testing**					
WBC (10^9/L)	NA	4.3	4.8	4.7	7.1
Haemoglobin (g/L)	NA	120↓	116↓	128↓	85↓

Platelet (10^9/L)	NA	55↓	32↓	29↓	52↓
RBC (10^{12}/L)	NA	3.99 ↓	3.97 ↓	4.32	3.03 ↓
Albumin (g/L)	NA	33.1 ↓	37.8↓	36.3↓	34.6 ↓
Globulin (g/L)	NA	41.1 ↑	44.5↑	43.0↑	32.6
Creatinine phosphokinase (umol/L)	NA	55.1	48.4	49.7	252.6 ↑
ESR	NA	32↑	56↑	19 ↑	35 ↑

"-"represents no indication; WBC: white blood cell; RBC: red blood cell; ESR: erythrocyte sedimentation rate.
